# 100 years of the theory of the gene: Thomas Hunt Morgan’s gene theory and the rise of modern genetics

**DOI:** 10.1038/s44319-026-00770-z

**Published:** 2026-04-04

**Authors:** Yongsheng Liu

**Affiliations:** Henan Institute of Science and Technology, Xinxiang, China

**Keywords:** Genetics, Gene Therapy & Genetic Disease, History & Philosophy of Science

## Abstract

In 1926, Thomas Hunt Morgan published *The Theory of the Gene*, which provided a mechanism for Mendel’s laws of inheritance, and paved the ground for modern genetics. It enabled numerous discoveries and new technologies, adding to our knowledge and putting more nuance to Morgan’s original theory.

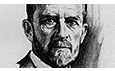

Thomas Hunt Morgan (Fig. 1) was born on September 25, 1866, the same year when Gregor Mendel (Fig. 2) published his landmark paper *Experiments on plant hybrids* that described the laws of inheritance. Like Mendel, Morgan is widely considered one of the most influential scientists in the history of genetics. One of his outstanding skills was his ability to choose the right people for his research teams. From 1909 onwards, he organized a brilliant group of researchers to study the problems of inheritance, first at Columbia University and later at the California Institute of Technology. The main contributions of Morgan and his school were to establish that the Mendelian inheritable elements, or genes, are parts of chromosomes, and the case for gene linkage and crossing-over. In 1926, Morgan published his cumulative work as a book, *The Theory of the Gene*, which is one of the classic texts of genetics and the dominant theory to explain the nature and location of genes (Morgan, 1926). He was awarded the Nobel Prize in Physiology or Medicine in 1933 for “his discoveries concerning the role played by the chromosomes in heredity.” Now, 100 years after the publication of Morgan’s gene theory, how does it hold up in light of both earlier theories on inheritance and novel insights from molecular biology?

**Figure 1:**
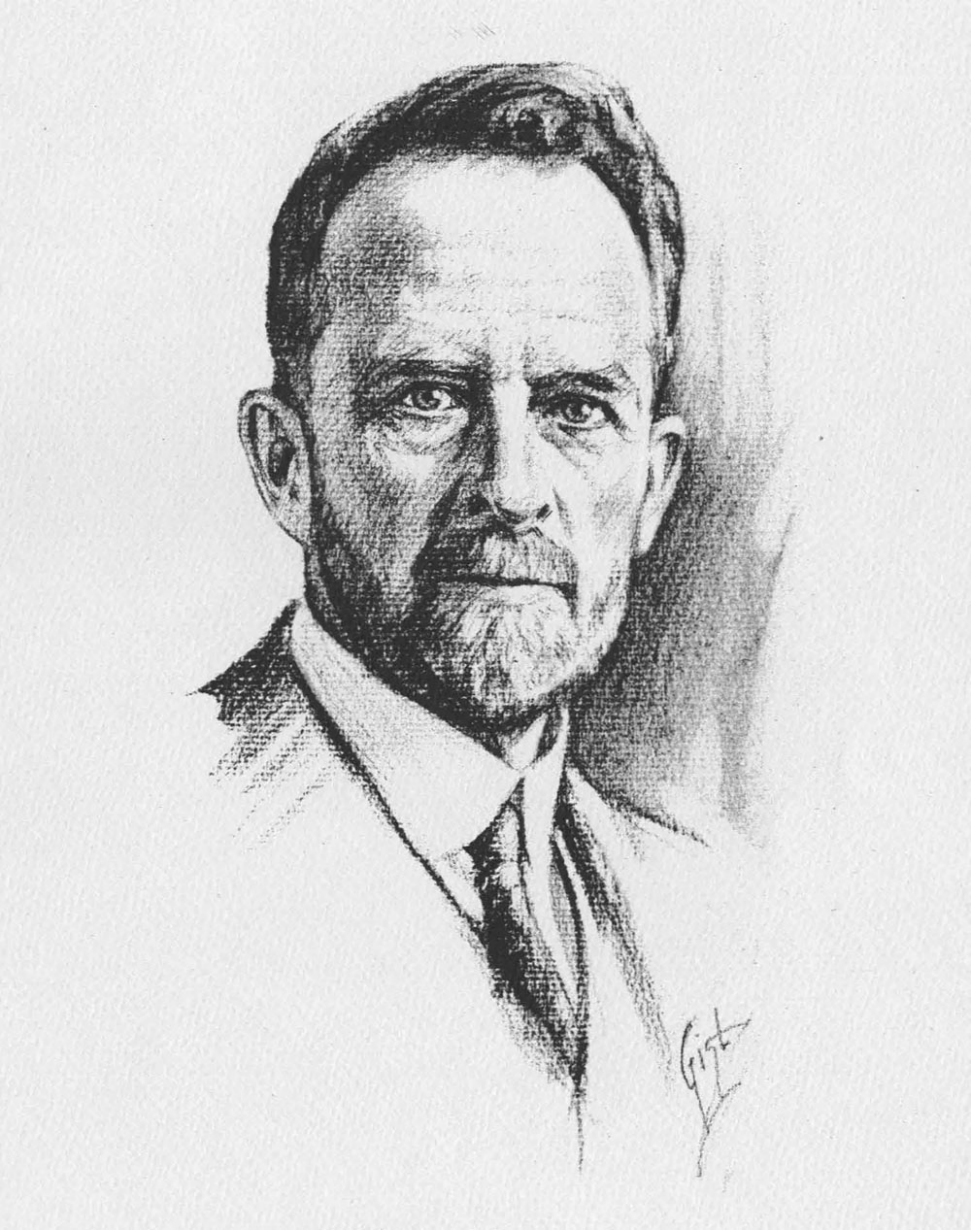
Thomas Hunt Morgan (1866–1945), sketch by Carl Rudolph Gist, 1931. Wikimedia Commons/Public Domain.

**Figure 2:**
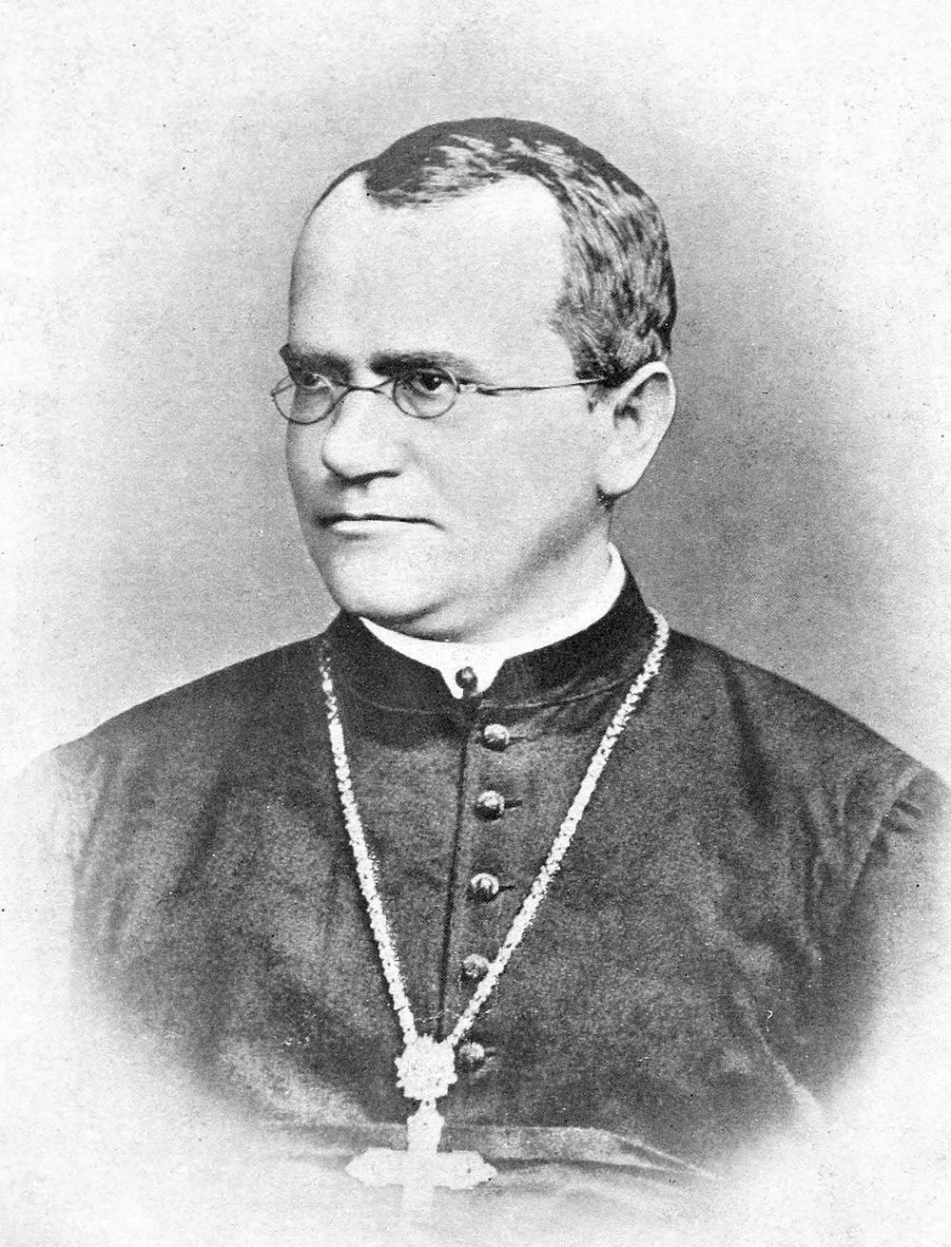
Gregor Mendel (1822–1884). Wikimedia Commons/Public Domain.

… Morgan is widely considered one of the most influential scientists in the history of genetics.

## The origin of the “gene”: from Darwin to Johannsen

**Figure 3:**
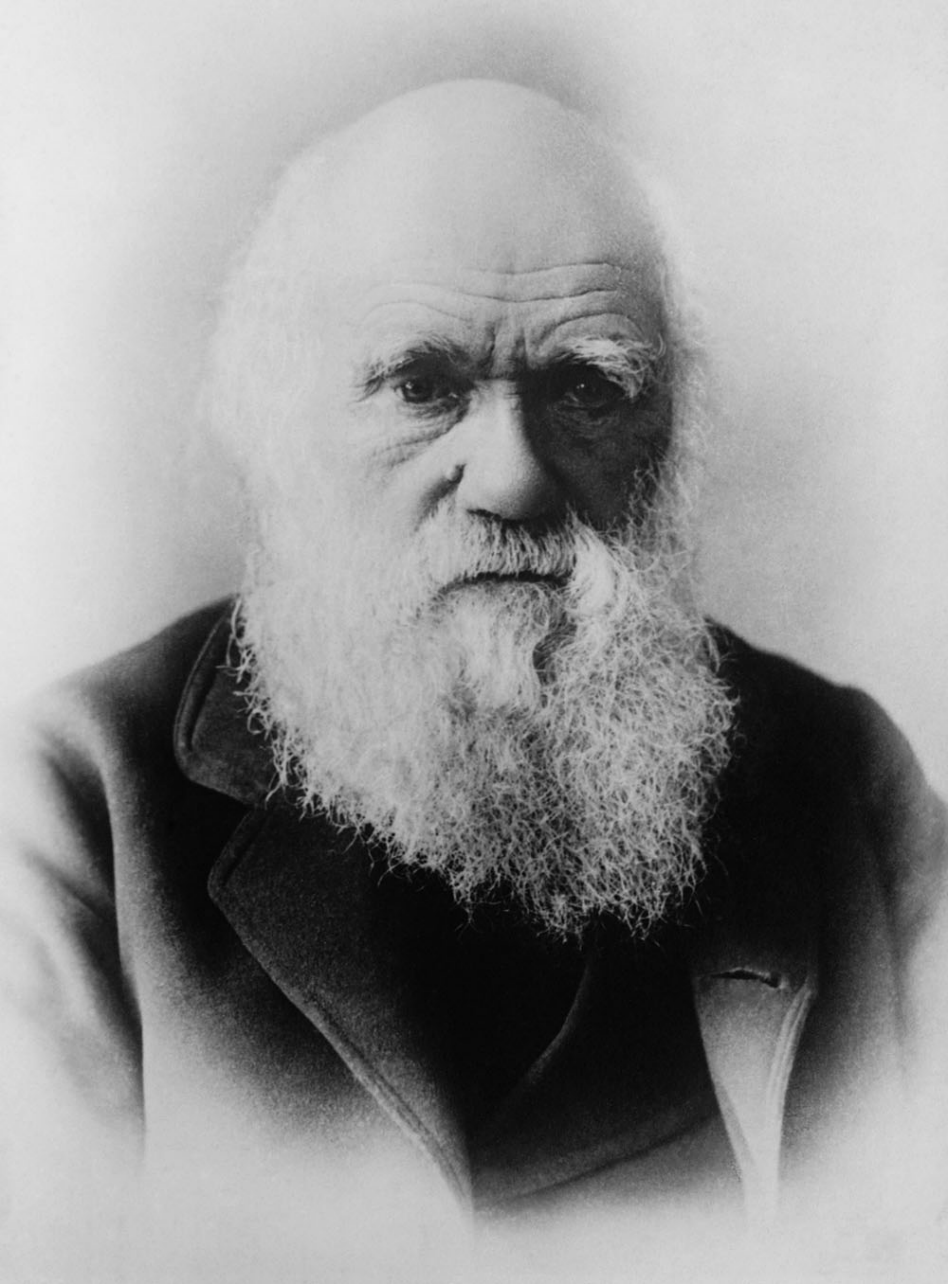
Charles Darwin (1809–1882), Elliott & Fry. Wikimedia Commons/Public Domain.

The central concept of genetics is the gene, the origins of which can be traced back to Charles Darwin (Fig. 3). In his *Essay of 1844*, Darwin described “germinal vesicles” as bearers of biological information that were “impressed with some power” to determine heritable characters. In *The Origin of Species*, he consistently used “element” to denote hereditary material. In 1868, Darwin published his Pangenesis hypothesis and used “gemmule” as a substitute for “germinal vesicle” and “element” (Darwin, 1868). He hypothesized that cells of an organism continually generate numerous gemmules that are modified by the conditions of life and by the use or disuse of organs and that are capable of self-multiplication, diffusion among cells, union with other gemmules and cells, and incorporation into sexual organs.

In 1889, Hugo de Vries modified Darwin’s Pangenesis and published *Intracellular Pangenesis*. He christened the inherited particle by a new term, “pangen”, which was derived from Darwin’s “Pangenesis”. Pangens, according to de Vries’ theory, were supposed to be transported only from the nucleus to other parts of the protoplast within a cell, and only from a cell to its daughter cells (de Vries, 1910). In 1909, Wilhelm Johannsen proposed “gen” as a simplification of “pangen”. In his words, “instead therefore of pangen (*das Pangen*) and pangens (*die Pangene*), we shall simply say gen (*das Gen*) and gens (*die Gene*)” (Cook, 1912). Johannsen used the term mostly in the German plural form “*Gene*”, which was adopted into English as the singular “gene”.

## From Darwin to the theory of the gene

In the chapter “Hybridism” of *The Origin of Species*, Darwin described that when two varieties of plants were crossed, one showed a “prepotency” over the other, which resembles Mendel’s concept of “dominance”. Mendel, in his attempts to discover the laws governing the distribution of parental characters amongst hybrid offspring, chose closely allied plant varieties separated by clearly defined and observable characters. The essence of his discovery is that inherited characters are independent of one another and that each is transmitted from parent to offspring as a separate unit. He formulated the laws of inheritance as an elegant mathematical formula to describe how different characters from the parents distribute among their descendants. At the time, Mendel could not know the physical nature of heritable elements and sstherefore did not distinguish between heritable characters and elements (Olby, 1979).

Mendel’s work remained largely unknown however until the turn of the century when Erich von Tschermak, Hugo de Vries and Carl Correns independently ‘rediscovered’ and verified his experimental results. In the meantime, August Weisman formulated the germ-plasm theory according to which only germ cells are the vehicles of inheritance whereas somatic cells are not involved. Weisman also stated that inheritance is a one-way road as somatic cells are generated by germ cells which are not affected by any of the changes that somatic cells undergo throughout life. This became known as the Weisman barrier—information cannot pass from soma to germ plasma and therefore to the next generation—effectively denying the inheritance of acquired characters as Jean-Baptiste Lamarck had proposed.

The first to suggest a relationship between physical elements and inheritable characters was Walter Sutton, who linked cytological observations of dividing germ cells with Mendel’s work. He proposed that Mendelian segregation of alleles took place during the meiotic separation of parental chromosomes (Sutton, 1902).

Although Morgan was initially skeptical of the role of chromosomes in mediating Mendelian inheritance, he eventually changed his views in light of accumulating empirical evidence. *The Theory of the Gene* extended Sutton’s theory and stated that Mendel’s inheritable characters refer to paired genes in the germinal cells. His theory thus provided the genotype-phenotype relationship that was missing from Mendel’s laws of inheritance: genes are integral parts of chromosomes, and specific genes are linked to specific hereditary characters.

Morgan further extended Mendel’s findings by proposing that genes were inherited as linkage groups. His statement that “the members of each pair of genes separate when the germ-cells mature” and “the members belonging to different linkage groups assort independently” beautifully confirmed Mendel’s first and second law.

Another of Morgan’s great contributions to genetics was his discovery of crossing-over, a term he invented to describe how paired chromosomes exchange genetic material during meiosis. This phenomenon not only explains the recombination of maternal and paternal genes but also allows Morgan’s team to specify the location of individual genes on chromosomes. If two genes were situated next to each other in the chromosome, a crossing-over event would include both genes with high probability, thereby accounting for the linkage groups he described in his theory. The farther apart the genes are, the less frequently would they be contained in the same linkage group. The relative frequency of crossing-over events between individual genes could therefore be used as an index to measure the distance between them to construct genetic maps. Morgan’s discovery of crossing-over and linkage groups formed the basis of a great number of genetic studies to identify phenotype-genotype relationships and construct genetic maps for a wide range of organisms during the 20th century (Whitehouse, 1963).

Morgan’s discovery of crossing-over and linkage groups formed the basis of a great number of genetic studies to identify phenotype-genotype relationships and construct genetic maps for a wide range of organisms…

## The gene theory in relation to mutation

Further elaborating on Darwin’s work, de Vries wrote: “for myself Pangenesis has always been the starting point of my inquiries; […] Especially is it this hypothesis which has led me to search for mutations in the field.” He posited that mutations could give rise to new species and considered this as the only mechanism by which evolutionary changes occur.

When Morgan became acquainted with de Vries’ work, he began to inquire how variation affects evolution by means of mutations. While de Vries had observed mutations in plants, Morgan selected an animal, the fruit fly. In 1909, he and his team started their investigation, and their remarkable assiduity in finding mutations made *Drosophila* become one of the best-known organisms genetically. Among others, they found that the “white eye” mutation was always inherited with the sex-determining X chromosome, providing further evidence to his theory that genes are located on chromosomes.

Morgan also stated that mutations are essential for studying the phenotype of a specific gene. In fact, this was the basic tenet for the modern synthesis of Darwin’s theory of evolution and Mendel’s work on heredity, which focused on the combination of natural selection and random mutations to drive evolution. Again, it laid the groundwork for modern genetics and its attempts to identify genotype-phenotype relationships by studying the effects of artificially introduced mutations.

While Morgan initially insisted that the majority of mutations are deleterious, and that “there is very little evidence that new species can often be produced in this way”, as experimental results accumulated, he eventually came to accept that mutations cause variety as the basis for natural selection. Morgan also agreed with Darwin’s original hypothesis that the causes of mutations and thereby variation come from the environment. This was shown later by Hermann Joseph Muller (1927), who discovered that mutations could be produced by artificial means such as X-rays, for which he was awarded the Nobel Prize in 1946.

Through his work on mutation and how these affect the function of individual genes and phenotypes, Morgan came to appreciate the complexity of how numerous genes interact to give rise to different phenotypes. Not just as a result of these insights, but also for moral reasons, he became a strong critic of the eugenics movement in the USA and its implicit racist views.

## DNA is the genetic material

It is striking that the three-year period between 1866 and 1869 saw Mendel’s publication of his classic paper, Darwin’s publication of his Pangenesis theory, and Friedrich Miescher’s discovery of nuclein; the term was replaced in 1899 by “nucleic acid”. Miescher already suspected the existence of a chemical code hidden in the hereditary material: “To me, the key to the problem of sexual reproduction is to be found in the field of stereo-chemistry. The ‘gemmules’ of Darwin’s Pangenesis are no other than the numerous asymmetrical carbon atoms of organic substances” (Olby and Posner, 1967). He proposed that hereditary information might be encoded in macromolecules, and that stereometrics changes might be the cause of mutation. Morgan (1926) proposed that the gene was probably a protein particle, about 0.2 microns in diameter, that is associated with the chromosome.

It is striking that the three-year period between 1866 and 1869 saw Mendel’s publication of his classic paper, Darwin’s publication of his Pangenesis theory, and Friedrich Miescher’s discovery of nuclein…

Soon thereafter, nucleic acids moved into focus as the candidate for the substance of heredity. In 1928, Frederick Griffith discovered a “transforming principle” which turned non-virulent bacteria into virulent ones (Griffith, 1928). Finally, in 1944, Oswald Avery, Colin MacLeod, and Maclyn McCarty showed in an elegant experiment that Griffith’s “transforming principle” was DNA. J. B. S. Haldane proposed a process of reciprocal complementary copying as a possible mechanism to explain gene replication prior to cell division (Pollock, 1976). In 1953, James Watson and Francis Crick published the double-helical model of the DNA molecule, which eventually explained not only how genetic information is encoded in its structure, but also Haldane’s process of complementary copying. In 1962, they and Maurice Wilkins received the Nobel Prize “for their discoveries concerning the molecular structure of nucleic acids and its significance for information transfer in living material”. In 1958, Matthew Meselson and Franklin Stahl demonstrated the semiconservative nature of DNA replication, by which each strand serves as a template for duplication. The knowledge about the structure of DNA finally allowed the cracking of the genetic code, for which Marshall Nirenberg, Har Gobind Khorana and Robert Holley received the Nobel Prize in 1968. It also led to the development of DNA sequencing in 1977 by Fredrick Sanger, who was awarded with his (second) Nobel Prize in Chemistry in 1980. Their work propelled genetics to the next wave of great discoveries.

## Transposable elements and horizontal gene transfer

Morgan considered the stability of the gene as one of the basic principles of the gene theory and understood genes as beads arranged in linear order on the chromosome. Later studies, however, put this notion in question showing that some genes are relatively stable, but others undergo conversion frequently. Genes may also be substantially stable in one breeding and unstable in another (Lindegren, 1956).

In the late 1940s, Barbara McClintock demonstrated that some genes frequently changed their position in the genome. She studied chromosome breakage in maize and found that a few genes do not have a fixed location but were moving within or between chromosomes triggering changes in gene expression. McClintock’s studies of these “transposons”, for which she received the Nobel Prize in 1983, showed that the genome does, in fact, undergo restructuring (McClintock, 1984). Today, it is well documented that transposable elements are surprisingly abundant and play a role in genome restructuring, gene expression, and development.

McClintock’s studies of these ‘transposons’, for which she received the Nobel Prize in 1983, showed that the genome does, in fact, undergo restructuring.

In 1948, cell-free nucleic acids were discovered for the first time in human blood. This discovery – and further evidence of the existence of extracellular DNA in the following decades – showed that many cell types release DNA molecules into their environment. In prokaryotes, this mechanism facilitates horizontal gene transfer and acts as a stabilizing ‘glue’ in biofilms. Neutrophils of the immune system release DNA to trap and kill pathogens, and small DNA molecules act as signaling molecules to regulate inflammation or immune responses. Free DNA from apoptosis can also be taken up by other cells, potentially contributing to cancer risk, while cancer cells, similar to eukaryotes, seem to exchange oncogenes or drug resistance genes via extrachromosomal circular DNA.

The existence of DNA and RNA outside the cell also provides a mechanism for “graft hybridization”, the formation of hybrids between distinct plant species by grafting. There has been increasing evidence for graft-induced genetic changes (Liu, 2006), and even entire nuclear genomes have been shown to move across the graft union, leading to the genesis of a new polyploid species (Fuentes et al, 2014).

## The rise of epigenetics

Morgan considered himself to be an embryologist rather than a geneticist. But the primary concern of the gene theory was transmissional genetics, making no attempt to explain development. Indeed, he commented that his gene theory “states nothing with respect to the way in which the genes are connected with the end-product or character”, arguing that the transmission of characters to offspring can be explained, “without reference to the way in which the gene affects the developmental process.”

Again, later discoveries expanded Morgan’s theory. Although most heritable information is indeed encoded in the nuclear genome, the discovery of cytoplasmic inheritance by Carl Correns in 1933 showed the transmission of maternal, non-chromosomal effects. In 1942, Conrad Waddington suggested “epigenetics” as the missing link between inheritance and development: “We might use the name ‘epigenetics’ for such studies, thus emphasizing their relation to the concepts, so strongly favorable to the classical theory of epigenesis, which have been reached by the experimental embryologists. We certainly need to remember that between genotype and phenotype, and connecting them to each other, there lies a whole complex of developmental processes” (Waddington, 1942). Waddington’s epigenetics was consistent with Darwin’s original views that developmental processes produce variation on which natural selection acts, proposing that inheritance, variation and development are tied together throughout the life of the organism. The discovery of the *Hox* gene in the 1980s—that controls developmental processes—inspired the field of Evolutionary Developmental Biology that studies how genetic changes during development affect the appearance of new phenotypes.

In 1942, Conrad Waddington suggested “epigenetics” as the missing link between inheritance and development…

Epigenetics is now defined as “the study of molecules and mechanisms that can perpetuate alternative gene activity states in the context of the same DNA sequence,” and is relevant to phenomena that have traditionally been attributed to the inheritance of acquired characters (Cavalli and Heard, 2019). While Darwin believed in the inheritance of acquired characters, although he accepted it as subordinate to natural selection, Morgan agreed with Weismann in denying the inheritance of acquired characters and made no attempt to elaborate his gene theory to account for it.

In recent years, there has been a growing body of experimental and epidemiological evidence for heritable changes induced by environmental factors, now included under the blanket term “transgenerational epigenetic inheritance” (Lindley et al, 2026). While the mechanisms are still elusive, studies point to chromatin modification and small RNAs as vectors of environmentally induced heritable changes (Chen et al, 2016; Posner et al, 2019).

## No theory should be final

Morgan maintained an open mind throughout his career and commented that his gene theory “need not and does not, of course, pretend to be final. It will, no doubt, undergo many changes and improvements in new directions.” He was one of the most influential proponents of Mendelism, and his gene theory provides the missing link between inheritable characteristics and inheritable elements along with a molecular mechanism for Mendelian inheritance. As any fruitful theory, it paved the way for further discoveries and insights by those who built upon Morgan’s work, further expanding it to explain many other phenomena in biology that could not be brought under Mendel’s laws (MacBride, 1937). As Darwin wrote: “In scientific investigations it is permitted to invent any hypothesis, and if it explains various large and independent classes of facts it rises to the rank of a well-grounding theory” (Darwin, 1868). As such, the theory of the gene has stood the test of time a century later. In the same vein, much of the knowledge and insights that have emerged from it have also validated Darwin’s theory of evolution as supported his Pangenesis theory.

As any fruitful theory, it paved the way for further discoveries and insights by those who built upon Morgan’s work, further expanding it to explain many other phenomena in biology that could not be brought under Mendel’s laws.

## Supplementary information

Peer Review File
