# Supplementary material for: 100 years of the theory of the gene: Thomas Hunt Morgan’s gene theory and the rise of modern genetics
Source: EMBO Rep. 2026 Apr 4;27(10):2517–21. doi: 10.1038/s44319-026-00770-z (PMC13219426; doi:10.1038/s44319-026-00770-z)
Supplement: Supplementary file 1 — Peer Review File [file 44319_2026_770_MOESM1_ESM.pdf]

EMBO reports

100 years of gene theory

Yongsheng Liu

Corresponding author(s): Yongsheng Liu (ysliu63@yahoo.ca)

Review Timeline:

Submission Date:	1st Feb 26
Accepted:	27th Mar 26

Editor: Holger Breithaupt

Transaction Report: This article was editorially reviewed.
